# Expression of growth factor receptors and targeting of EGFR in cholangiocarcinoma cell lines

**DOI:** 10.1186/1471-2407-10-302

**Published:** 2010-06-18

**Authors:** Ling Xu, Martin Hausmann, Wolfgang Dietmaier, Silvia Kellermeier, Theresa Pesch, Manuela Stieber-Gunckel, Elisabeth Lippert, Frank Klebl, Gerhard Rogler

**Affiliations:** 1 Department of Internal Medicine I, University of Regensburg, 93042 Regensburg, Germany; 2 Department of Medical Oncology, Peking University First Hospital, 100034 Peking, China; 3 Clinic of Gastroenterology and Hepatology, Department of Internal Medicine, University Hospital of Zürich, 8091 Zürich, Switzerland; 4 Department of Pathology, University of Regensburg, 93042 Regensburg, Germany

## Abstract

**Background:**

Cholangiocarcinoma (CC) is a malignant neoplasm of the bile ducts or the gallbladder. Targeting of growth factor receptors showed therapeutic potential in palliative settings for many solid tumors. The aim of this study was to determine the expression of seven growth factor receptors in CC cell lines and to assess the effect of blocking the EGFR receptor *in vitro*.

**Methods:**

Expression of EGFR (epithelial growth factor receptor), HGFR (hepatocyte growth factor receptor) IGF1R (insulin-like growth factor 1 receptor), IGF2R (insulin-like growth factor 2 receptor) and VEGFR1-3 (vascular endothelial growth factor receptor 1-3) were examined in four human CC cell lines (EGI-1, HuH28, OZ and TFK-1). The effect of the anti-EGFR-antibody cetuximab on cell growth and apoptosis was studied and cell lines were examined for *KRAS *mutations.

**Results:**

EGFR, HGFR and IGFR1 were present in all four cell lines tested. IGFR2 expression was confirmed in EGI-1 and TFK-1. No growth-inhibitory effect was found in EGI-1 cells after incubation with cetuximab. Cetuximab dose-dependently inhibited growth in TFK-1. Increased apoptosis was only seen in TFK-1 cells at the highest cetuximab dose tested (1 mg/ml), with no dose-response-relationship at lower concentrations. In EGI-1 a heterozygous *KRAS *mutation was found in codon 12 (c.35G>A; p.G12D). HuH28, OZ and TFK-1 lacked *KRAS *mutation.

**Conclusion:**

CC cell lines express a pattern of different growth receptors *in vitro*. Growth factor inhibitor treatment could be affected from the *KRAS *genotype in CC. The expression of EGFR itself does not allow prognoses on growth inhibition by cetuximab.

## Background

Cholangiocarcinoma (CC) is a malignant neoplasm arising from the biliary epithelium. Most cases of CC occur sporadically and the exact aetiology is still unknown [1]. Chronic inflammation and biliary duct cell injury induced by the obstruction of bile flow are two of the main conditions responsible for the development of CC [2]. As yet complete surgical resection is the only curative treatment for CC. Potential for resection depends on the location and the stage of the tumor [3]. Commonly, more than 60% of CC patients have tumors not treatable by resection [4]. Patients with an operable tumor only have a 5-year median survival rate of 9-18% for proximal biliary lesions and 20-30% for more distal tumors [5]. Chemotherapy has been used in an attempt to control disease as well as to improve survival and quality of life in patients with irresectable, recurrent or metastatic CC [6]. Chemotherapy versus best supportive care (BSC) was compared in a randomized study including both CC and pancreatic carcinoma [7]. Patients in the chemotherapy group had an improved quality of life compared to those in the BSC group.

Most chemotherapies applied for CC to date are based on 5-fluorouracil (5-FU) or gemcitabine. Median survival times reported for palliative chemotherapy range from 4.6 to 15.4 months, which are far from desirable [6]. Radiotherapy is also insufficiently effective in treating CC [8].

EGFR and the EGF-family of peptide growth factors play a central role in the pathogenesis and progression of different carcinoma types [9,10]. Manifold actions for other growth factors and their receptors systems have been described in cancer, e.g. IGF (insulin-like growth factor)/IGFR system and HGF (hepatocyte growth factor)/HGFR systems [11-13]. Based on expression data of growth factor receptors, therapeutic targeting of these receptors has been attempted in tumor patients.

Targeting of two of these systems, EGFR and VEGFR has shown potential [14]. The agents which target EGFR can be classified into two groups: tyrosine kinase inhibitors (TKIs), such as gefitinib and erlotinib, and monoclonal antibodies, such as cetuximab or panitumumab. In particular, the use of cetuximab in gastrointestinal malignancies has reached an advanced stage of clinical development. It has been approved by the Food and Drug Administration (FDA) for the treatment of patients with EGFR-expressing metastatic colorectal cancer. Cetuximab induces consistent response rates as a single agent (approximately 10% to 15% overall response rate) and in combination with chemotherapy in metastatic colorectal carcinoma patients [15]. The mutation status of the *KRAS *gene affects the response of cetuximab. Patients with a colorectal tumor bearing mutated *KRAS *did not benefit from cetuximab, whereas patients with a tumor bearing wild-type *KRAS *did [16]. Further non-gastrointestinal indications for cetuximab include SCCHN (squamous cell carcinoma of the head and neck), and NSCLC (non-small cell lung cancer). Agents targeting IGF/IGFR and HGF/HGFR systems are also in development [11,17,18].

No conclusive data is available on the effect of these new therapeutic strategies in CC. Knowledge about the expression of growth factor receptors may guide the development of new therapeutic strategies in CC. Therefore, the aim of this study was to determine the expression of EGFR, IGF1R, IGF2R, HGFR and VEGFR1-3 in four human CC cell lines. In addition, the effect of the monoclonal anti-EGFR antibody cetuximab on cell growth and apoptosis in two of these cell lines was studied.

## Methods

### Cell culture

Human CC cell lines EGI-1 and TFK-1 were purchased from DSMZ (German Collection of Microorganisms and Cell Cultures, Human and Animal Cell Lines, Braunschweig, Germany). Human CC cell lines HuH28 and OZ were obtained from HSRRB (Japan Health Sciences Foundation, Health Science Research Resources Bank, Tokyo, Japan). EGI-1 cells were cultured in Dulbecco's MEM medium (Sigma, Munich, Germany) with 10% fetal bovine serum (FBS), MEM essential and non-essential amino acids (PAA, Pasching, Austria); HuH28 cells were cultured in RPMI1640 medium with 10% FBS, MEM essential and non-essential amino acids, sodium pyruvate solution (PAA) and MEM vitamins (Biochrom, Berlin, Germany), OZ cells were cultured in William's E medium (PAN Biotech GmbH, Aidenbach, Germany) with 10% FBS and TFK-1 cells were cultured in RPMI1640 medium (Sigma) with 10% (FBS, PAN Biotech GmbH). EGI-1 cells were cultured at 37°C and 10% CO_2 _atmosphere. HuH28, OZ and TFK-1 cells were cultured at 37°C and 5% CO_2 _atmosphere.

### RT-PCR

Total RNA was extracted from cells using the RNeasy mini-Kit (Qiagen, Hilden, Germany) according to the manufacturer's instructions. RNA was synthesised to first strand cDNA using the Reverse Transcription System (Promega, Madison, WI, USA) following the manufacturer's protocol (15 min reaction at 42°C). The reactions were performed in a TRIO Thermoblock (Biometra, Goettingen, Germany). To test presence and integrity of the cDNA PCR for five different housekeeping genes was performed (2 K Clathrin 500 bp, 6 K Clathrin 500 bp, GAPDH 540 bp, 3'-actin 720 bp and 5'-actin 1 kb from the Gene Checker™ Kit, Invitrogen, Leek, The Netherlands). The PCR comprised 25 cycles with denaturing at 94°C for 30 s, annealing at 55°C for 30 s and extension at 72°C for 30 s. PCR primers for growth factor receptors were synthesized by TIB MOLBIOL (Berlin, Germany). cDNA sequences were obtained from the NCBI GenBank. Primer sequences, annealing temperatures and PCR product lengths are given in Table 1. PCR reactions were performed with Advantage^® ^cDNA Polymerase Mix (Clontech, Saint-Germain-en-Laye, France). 1.2% agarose gel (Invitrogen, Karlsruhe, Germany) was used for loading of DNA products and electrophoresis. 100 bp DNA Ladder Plus (Gene Ruler™, Fermentas, St. Leon-Rot, Germany) was used as the standard. Growth factor receptor mRNA was only determined qualitatively, no quantitative PCR was performed.

**Table 1:** Primer sequences, expected PCR product length and annealing temperature (temp) for growth factor receptor RT-PCR

	Primer	Product (bp)	Temp (°C)
**EGFR**	forward: 5'- ATG TCC GGG AAC ACA AAG AC -3' (2648-2667)	351	54
			
**NM_005228.3**	reverse: 5'- TTC CGT CAT ATG GCT TGG AT - 3' (2979-2998)		

**IGFR1**	forward: 5'- ACC CGG AGT ACT TCA GCG CT -3' (2980-2999)	230	50
			
**NM_000875.3**	reverse: 5'-CAC AGA AGC TTC GTT GAG AA -3' (3190-3209)		

**IGFR2**	forward: 5'- GCT GAC CAC TTG CTG TAG GAG AAG -3' (7162-7185)	220	50
			
**NM_000876.2**	reverse: 5'- ATC CTC ACT GTC CTG GTC ATC CC -3' (7359-7381)		

**HGFR**	forward: 5'- GGT CAA TTC AGC GAA GTC CT -3' (1481-1500)	242	50
			
**NM_000245.2**	reverse: 5'- TTC GTG ATC TTC TTC CCA GTG -3' (1702-1722)		

**VEGFR1**	forward: 5'- TCG TGT AAG GAG TGG ACC ATC A -3' (1218-1239)	470	50
			
**NM_002019.3**	reverse: 5'- GCC AGA ACC ACT TGA TTG TAG G -3' (1690-1669)		

**VEGFR2**	forward: 5'- GCG GTG ATT GCC ATG TTC TTC -3' (2616-2636)	550	50
			
**NM_002253.2**	reverse: 5'- CGC CGT TTC AGA TCC ACA GG -3' (3187-3168)		

**VEGFR3**	forward: 5'- GGC AGC ATG GAG ATC GTG AT -3' (2393-2412)	380	50
			
**NM_182925.4**	reverse: 5'- GGT TGC CGA TGT GAA TGA GG -3' (2795-2776)		

### Western blot

Cells were washed with ice-cold phosphate buffered saline (PBS) two times, lysed in CHAPS lysis buffer (Sigma, Munich, Germany), supplemented with DTT (Sigma, Munich, Germany) for 30 min on ice, then sonicated and centrifuged for 5 min at 13.200 rpm. The supernatant was used for Western blot. Protein samples were separated on 4-12% Tris-Glycine gels (Invitrogen, Novex^®^) with TrisGly running buffer (Novex^®^), transferred onto a nitrocellulose membrane and incubated with the following specific antibodies: polyclonal rabbit anti-human EGFR antibody (#EGFR(1005):SC-03, Santa Cruz Biotechnology, Santa Cruz, California, final concentration 0.5 μg/ml), mouse monoclonal anti-human HGFR (#MAB3582, R&D Systems, Wiesbaden-Nordenstadt, Germany) antibody, mouse monoclonal anti-human IGF1R antibody (#MAB391, R&D Systems), goat anti-human IGF2R (#AF2447, R&D Systems) antibody, mouse monoclonal anti-human VEGFR-1 antibody (IgG1, DM 3504, Acris Antibodies, Hiddenhausen, Germany, final concentration 1 μg/ml), mouse monoclonal anti-human VEGFR-2 antibody (IgG1, DM 3503, Acris Antibodies, final concentration 2 μg/ml) and mouse monoclonal anti-human VEGFR-3 antibody (IgG, DM 3512P, Acris Antibodies, final concentration 0.5 μg/ml). Horseradish peroxidase-conjugated antibodies were applied as secondary antibodies: goat anti-rabbit (#SC2004, Santa Cruz Biotechnology, final concentration 0.08 μg/ml) was used for the detection of EGFR. Goat anti-mouse antibody (#SC2005 Santa Cruz Biotechnology) for IGF1R and HGFR. Donkey anti-goat antibody (#SC2020, Santa Cruz Biotechnology) for IGF2R. Goat anti-mouse antibody were used as secondary antibodies for the detection of VEGFR1-3 (#A0168, Sigma, 1:5000 dilution). Immune complexes were visualized by enhanced chemiluminescence (ECL Plus Kit, Amersham Biosciences, Braunschweig, Germany).

### Immunohistochemistry (IHC)

The following primary antibodies were used for IHC: mouse monoclonal anti-human EGFR antibody (IgG, #E3138, Sigma), mouse monoclonal anti-human HGFR antibody (IgM, #MONX10170, Monosan, Am Uden, The Netherlands), mouse monoclonal anti-human IGF1R antibody (IgG1, #MAB391) and goat anti-human IGF2R antibody (#AF2447 both from R&D Systems). Mouse monoclonal anti-human VEGFR1 antibody (IgG1, #MAB321, R&D Systems), goat polyclonal anti-human VEGFR2 antibody (IgG, #AF357, R&D Systems) and mouse monoclonal anti-human VEGFR-3 antibody (IgG1, #MAB3491, R&D Systems). Mouse IgG (#M5284, Sigma), mouse IgM (#X0942) and goat serum (#X0907, DakoCytomation, Hamburg, Germany) were used as isotype controls. Biotinylated goat anti-mouse immunoglobulins (#E0433, DakoCytomation) or biotin-SP-conjugated mouse anti-goat-IgG antibodies (#205-065-108, Jackson ImmunoResearch, Newmarket, England) were used as secondary antibodies.

For IHC analysis, cells were cultured in 4-chamber slides for 3-4 days, and fixed with 3.7% formaldehyde for 15 min. IGF1R was studied with the APAAP method. Fixed cells were rinsed with TBS for 5 min and incubated in TBS with 10% FBS for 60 min at room temperature. Primary antibodies were applied in humid chambers for 60 min. The APAAP kit was used according to the manufacturer's instructions (DakoCytomation).

EGFR, HGFR, and IGF2R were stained by the standard ABC procedure. After fixation and washing as described above, 0.3% hydrogen peroxide was used to block endogenous peroxidase. PBS with 1% FBS was applied as blocking solution. Cells were incubated with primary antibodies, biotinylated secondary anibodies and an avidin-biotin-peroxidase complex (Vectastain PK-6100, Linaris, Wertheim-Bettingen, Germany). Samples were dyed with substrate kits for peroxidase (DAB SK-4100 yielding a brown stain, VectorRed SK-4800 yielding a red stain, both from Vector^®^, Linaris).

No quantification of protein expression was performed - nor in the immunohistochemistry nor in the Western blot experiments.

### Analysis of *KRAS *mutations

DNA was isolated from the four human CC cell lines EGI-1, HuH28, OZ and TFK-1 cell lines using a kit (#51306, DNA purification Kit for genomic DNA from tissue blood and body fluids, Qiagen) according to the supplied protocol. Mutation analysis was done by direct sequencing of PCR amplified *KRAS *exon 2 containing codon12 and 13. PCR amplification reactions were carried out in final volume of 30 μl containing 200 μM of each dNTP, 0.3 μM of each primer, 50-100 ng genomic template DNA, and 0.6 U Taq DNA polymerase in 1 × PCR buffer (Fermentas, Germany) using a MJ Research Thermocycler (PTC100, MJ Research, Watertown, MA). For amplification an initial denaturation step (94°C/2 min) was followed by 35 rounds of thermal cycles (94°C/1 min, 55°C/1 min, and 72°C/1 min) and a final elongation at 72°C for 8 min.

The KRAS PCR primer sequences were 5'- TAA GGC CTG CTG AAA ATG AC -3' (KRAS-U) and 5'- AAA CAA GAT TTA CCT CTA TTG TTG GA -3' (KRAS-D). PCR products were purified using the QIAquick PCR purification kit (Qiagen) according to the supplier's protocol. DNA sequencing was performed using the ABI Prism Big Dye Terminator Cycle Sequencing Kit 1.1 (Applied Biosystems, Weiterstadt, Germany) and an ABI 3100 AVANT capillary electrophoresis system (Applied Biosystems) according to the manufacturer's instruction.

### Cell culture for cetuximab experiments and *in vitro *growth inhibition

**Figure 1:**
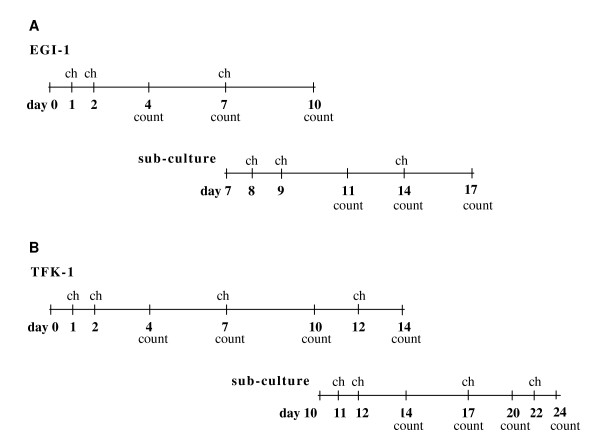
**Sub-culturing of CC cell lines**. Medium changes (ch) and time points for cell counting (count) for EGI-1 (A) and TFK-1 (B). Effects of cetuximab on growth of human CC cell lines was determined at the indicated time points. Apoptosis was observed by PI staining at the end of the sub-culture period.

To evaluate the effects of cetuximab on cell growth 1 × 10^4 ^EGI-1 and 1.5 × 10^4 ^TFK-1 cells were plated in 6-well cell culture plates. Medium was supplemented with penicillin 100 U/ml (PAA), streptomycin 0.1 mg/ml (PAA), ciprofloxacin 0.8 mg/ml (Ciprobay^® ^200, Bayer Schering Pharma AG, Leverkusen, Germany), gentamycin 50 μg/ml (PAA) and amphotericin B 1 μg/ml (Bristol-Myers-Squibb, Munich, Germany). Cetuximab was purchased from Merck Pharma GmbH (Erbitux^®^, Darmstadt, Germany). Cetuximab was diluted in medium, with final concentrations of 0.1 μg/ml, 1.0 μg/ml, 10 μg/ml, 100 μg/ml and 1000 μg/ml. Time points for sub-culture of cells and medium changes are given in figure 1. At times indicated, cells were harvested, stained with trypan blue (Biochrom AG, Berlin, Germany) and counted with an improved Neubauer counting chamber (Faust, Schaffhausen, Switzerland).

### Flow cytometry analysis of apoptosis

Apoptosis was observed by fluorescence activated cell sorting (FACS) analysis. At indicated time points cells were trypsinized, washed twice with Ca^2+^/Mg^2+ ^free PBS and fixed in 70% methanol. After treatment with RNase A (Qiagen), cells were incubated at 4°C in the dark with propidium iodide (PI) staining solution (Sigma). After 20 minutes of incubation, apoptosis rate was determined by flow cytometry utilizing a Coulter^® ^EPICS^® ^XL-MCL™ (Coulter, Immunotech, Krefeld, Germany) equipped with an argon ion laser with an excitation power of 15 mW at 488 nm. The fluorescence of cells was collected on a four decade log scale through forward light scatter (FSC) and linear scale through right angle scatter (SSC). Fluorescence for PI was collected at 620 nm (FL3). Analysis gates were set around debris and intact single cells on a PI versus AUX dot plot. Data were analyzed by the ExpoTM32 ADC program (Beckman Coulter, Krefeld, Germany).

### Statistics

All experiments were performed in triplicate. Data are expressed as mean ± s.d. Statistical analysis was performed using the Student's *t *test. Differences were considered significant with a *P *value of < 0.05. Curve progression of cell growth in cetuximab experiments was calculated with SPSS version 13·0.1 (Apache Software Foundation, Forest Hill, MD, USA). Differences were considered significant at a *P*_GLM_-value of < 0.001.

## Results

### mRNA expression of growth factor receptors in human CC cell lines

**Figure 2:**
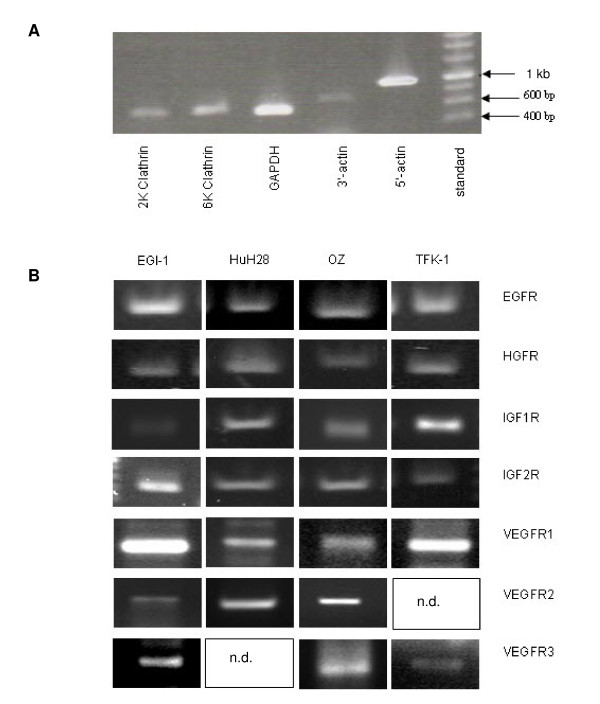
**RT-PCR for housekeeping genes and growth factor receptors**. A) PCR analysis to ensue the presence and integrity of five housekeeping genes from TFK-1, representative for all cell lines used. B) mRNA expression of growth factor receptors in four CC cell lines (n. d. = not detected).

The four CC cell lines were cultured as described. RNA was isolated and the integrity of mRNA was verified by RT-PCR with the Gene Checker™ kit proving presence and integrity (figure 2A). Negative control reactions were performed without cDNA. Growth factor receptor mRNA was analyzed in the CC cell lines. EGFR, HGFR, IGF1R, IGF2R and VEGFR1 were detected in all four cell lines (figure 2B). VEGFR2 was found in EGI-1, HuH28 and OZ but not in TFK-1. VEGFR3 RT-PCR was successful with cDNA from EGI-1, OZ and TFK-1 but not with cDNA from HuH28.

### Protein expression of growth factor receptor in human CC cell lines

**Figure 3:**
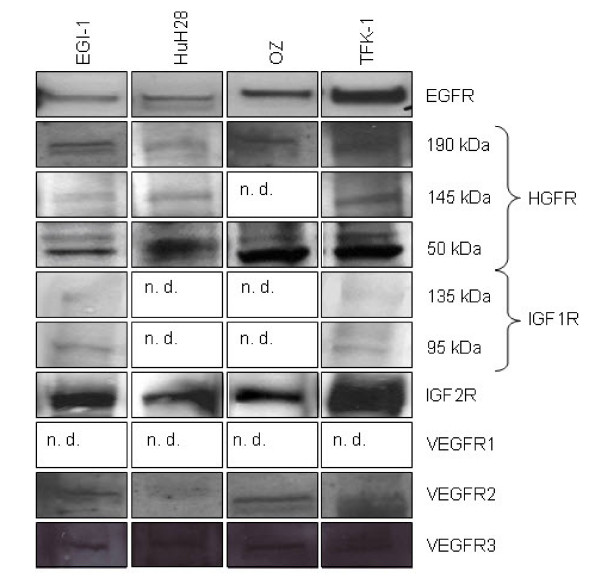
**Western blot for growth factor receptors from different CC cell lines**. EGFR, HGFR, IGF2R, VEGFR2 and 3 was detected in EGI-1, HuH28, OZ and TFK-1. IGF1R was visualized in EGI-1 and TFK-1. VEGFR1 was not in the investigated cell lines (n. d. = not determined).

To investigate whether mRNA expression was followed by translation into protein we performed Western blots with lysates from EGI-1, HuH28, OZ and TFK-1. The 170 kDa EGFR protein was detectable in all four human CC cell lines (figure 3). The heterodimeric mature form of the 190 kDa HGFR protein is processed to a 145 kDa and 50 kDa subunit. In EGI-1, HuH28 and TFK-1 the 145 kDa β chain was detected. The 50 kDa α chain was present in all four cell cultures. Hence, HGFR was considered to be expressed in all studied CC cell lines. Two bands of the heterotetrameric transmembrane protein IGF1R protein were found in EGI-1 and TFK-1 representing the two alpha-subunits of 135 kDa and two beta-subunits of 95 kDa (figure 3). IGF2R protein (300 kDa) was found in all of the four cell lines with strong immunoreactive bands. No evidence was seen for VEGFR1 protein (180 kDa) expression. The 200 kDa VEGFR2 band was clearly detectable in EGI-1 and OZ. A weak signal was obtained for HuH28. VEGFR3 protein (195 kDa) was determined in EGI-1, HuH28 and OZ. A faint signal could be identified in TFK-1.

### Growth factor receptor immunohistochemistry

**Figure 4:**
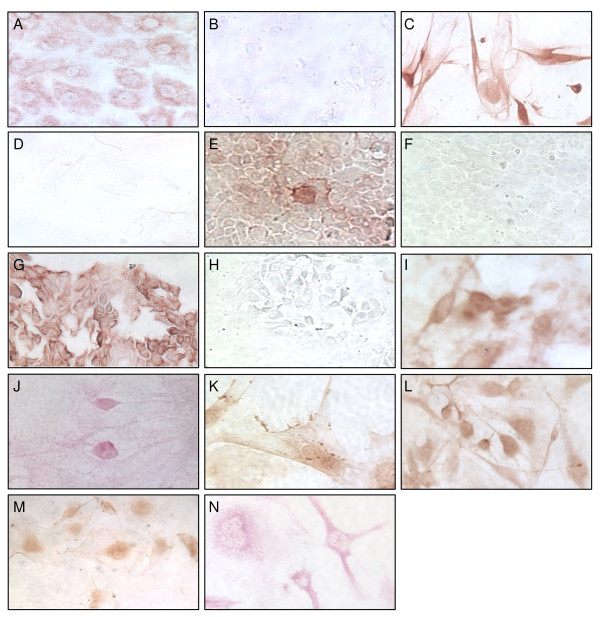
**IHC for growth factor receptors on different CC cell lines**. Immunostaining of EGFR on EGI-1 (A), HuH28 (C), OZ (E) and TFK-1 (G) with the according isotype staining (B, D, F and H). Immunostaining of HGFR (I), IGF1R (J), IGF2R (K), VEGFR1 (L), VEGFR2 (M) and VEGFR3 (N). IHC revealed expression of the growth factor receptors tested in all human CC cell lines. ABC procedure (A-I, K-M) and APAAP method (J and N). Original magnification × 200.

As mRNA and protein expression of the investigated growth factor receptors were confined to EGI-1, HuH28, OZ and TFK-1 by RT-PCR and Western blot, we confirmed protein expression in CC cell lines by IHC. Cells were grown on glass slides and analyzed at different states of confluence. Anti-EGFR antibody and the corresponding secondary antibody was applied and visualized by the standard ABC procedure. DAB substrate dye resulted in a brown reaction product. Immunostaining for EGFR demonstrated a clear protein expression in EGI-1 and TFK-1 (figure 4A and 4C). No detectable staining was obtained when a mouse IgG1 isotype was used as negative control (figure 4B and 4D). Staining of HuH28 and OZ yielded a diffuse brown color (figure 4E and 4G) as compared to isotype control (figure 4F and 4H).

Anti-HGFR, -IGF2R, -VEGFR1 and 2 were applied with secondary antibodies. An intensive brown staining was demonstrated using ABC and DAB for the growth factor receptors as shown for HuH28 representative for all four cell lines (figure 4I, K, L, M). No detectable staining was obtained when isotype control antibodies were used as negative control (not shown). IGF1R and VEGFR3 were visualized by the APAAP procedure and dyed with VectorRed substrate resulting in a clear red reaction product (figure 4J and 4N) compared to isotype control (not shown).

### Effects of cetuximab on growth of human CC cell lines

**Figure 5:**
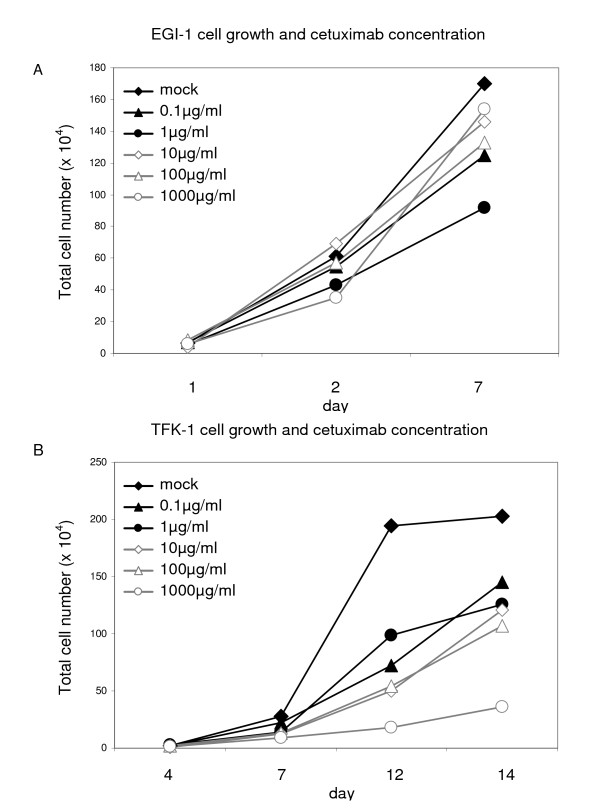
**Effects of cetuximab on growth of human CC cell lines**. No growth inhibition was observed for EGI-1 (A). Anti-EGFR antibody decreased growth of TFK-1 (B).

EGFR mRNA expression was determined by RT-PCR in EGI-1, HuH28, OZ and TFK-1 and further confirmed by Western blot and IHC. As Ras activation is likely to promote tumor cell proliferation to assess whether successful inhibition of the EGFR signaling pathway is associated with *KRAS *mutations cell lines were examined for *KRAS *gene mutations. To confirm mutated *KRAS *alleles, we extracted the corresponding genomic DNA and sequenced the *KRAS *locus. In EGI-1 a heterozygous mutation was found in codon 12 (c.35G>A; p.G12D). Codon 12 mutations usually lead to *KRAS *activation in colorectal cancer cell lines, but the activation status was not formally tested in EGI-1 [19]. HuH28, OZ and TFK-1 were confirmed to display the wild-type allele. This evidence suggests that in HuH28, OZ and TFK-1 no constitutive activation of the *KRAS *gene is present (data not shown). Two cell lines carrying either wild-type or mutated *KRAS *were chosen to test the effect of cetuximab on cell growth and apoptosis. We selected EGI-1 and TFK-1 for treatment with increasing concentrations of cetuximab (final concentrations 0.1-1000 μg/ml). The fast-growing cells were plated in 6-wells, supplied with sufficient media and sub-cultured to avoid confluence (figure 1A). Cells were counted with an improved Neubauer chamber at day 4, 7 and 10 (figure 5A) and day 11, 14 and 17 of sub-culture (not shown). Within 10 days non-stimulated EGI-1 proliferated from 1 × 10^4 ^to 1.7 × 10^5 ^cells. Under stimulation with cetuximab they grew to 1.5 × 10^5^-0.9 × 10^5 ^cells. Cetuximab showed no dose-dependent effect on cell growth in EGI-1. No growth inhibition was observed between initially seeded and sub-cultured EGI-1.

TFK-1 were counted at day 4, 7, 10 and 14 (figure 5B) and day 14, 17, 20 and 24 of sub-culture (not shown). Within 14 days non-stimulated TFK-1 proliferated from 1.5 × 10^4 ^to 2.3 × 10^5 ^cells. In contrast to EGI-1, TFK-1 displayed a dose-dependent inhibition of cell growth exerted by cetuximab. Stimulation with the anti-EGFR antibody decreased growth to 1.4 × 10^5 ^cells with 0.1 μg/ml cetuximab up to 0.3 × 10^5 ^cells with 1000 μg/ml. The same result was found in the sub-cultured cells.

### Effects of cetuximab on apoptosis of human CC cell lines

**Figure 6:**
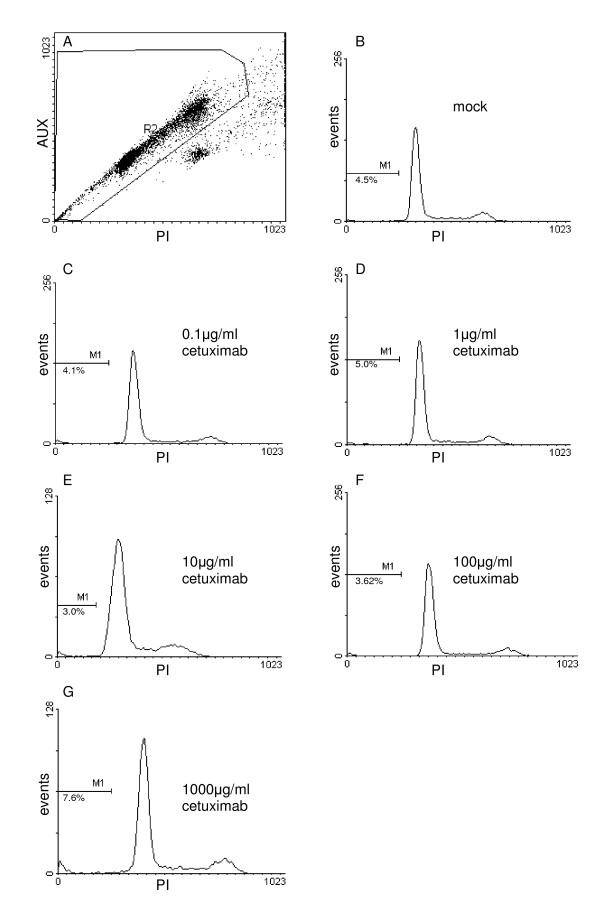
**Effects of cetuximab on apoptosis of TFK-1 cells**. Single cell gate (A) and PI staining after 24 days of culture (B-G). Cetuximab mediates apoptotic effects in TFK-1 cells at 1000 μg/ml only.

Stimulation with cetuximab showed no dose-dependent effect on cell growth in EGI-1 but a significant inhibition of cell growth in TFK-1 in both the initially seeded and sub-cultured cells. To assess whether cetuximab stimulation results in apoptotic effects fragmented DNA was measured by flow cytometry after PI staining. The percentages of apoptotic cells are depicted by the sub-G1 peak (M1) of fragmented DNA in figure 6. Treatment of cultured EGI-1 with cetuximab did not increase the number of apoptotic cells (not shown). An increase of apoptosis of from 4.5% to 7.6% in the initially seeded cells and from 7.2% to 17.6% in the sub-cultured TFK-1 was observed at the highest cetuximab concentration (1000 μg/ml). These data indicate that cetuximab mediates apoptotic effects in TFK-1 cells at high doses.

## Discussion

In recent years, the role of EGFR in CC has been the focus of several studies. EGFR expression was examined in 20 surgically resected liver tissues with CC by IHC and 25% (5/20) were EGFR-positive suggesting that this growth hormone receptor is associated with CC [20]. Nonomura et al. found EGFR in 32% of cases with intrahepatic CC by immunocytochemistry and reported some cases of co-expression with EGF, suggesting autocrine growth stimulation [21]. Another group analyzed the role of EGFR in intrahepatic CC [22]. EGFR expression was found to be correlated with frequency of lymph node metastases, aberrant p53 expression, proliferative activity and differentiation of the carcinoma. In a recent publication on EGFR expression in 236 cases of CC EGFR expression was a significant prognostic factor and also a risk factor for tumour recurrence in intrahepatic CC [23]. These results confirm that EGFR expression is associated with tumour progression.

EGFR has been attributed an important role in carcinogenesis of several tumor types and EGFR inhibitors are currently used in treatment of some of them [24-28]. Yoon et al. reported that EGF stimulation increased cell growth in CC cells [29]. The effect was significantly diminished by EGFR kinase inhibitors. A growth inhibitory effect of cetuximab has demonstrated in several cell lines of non-CC origin, e.g. in squamous cell [30], colon [31], head and neck [32], non-small cell lung [28], prostate [33], renal carcinoma [27] and glioblastoma [34]. It has been reported that the monoclonal anti-EGFR-antibody cetuximab was active against various tumors including colorectal [24], head and neck [25], non-small cell lung [28], prostate [26] and renal cancer [27]*in vivo*. Sprinzl et. al. described the treatment of a patient with a non-resectable CC in a case report [35]. Combination of cytotoxic chemotherapy together with cetuximab showed promising efficacy. Blocking EGFR on CC cells could represent a therapeutic approach in respect to survival and quality of life [35]. A recent publication assessed the efficacy of cetuximab in the palliative treatment of patients with intrahepatic CC unresponsive to first-line gemcitabine-oxaliplatin pretreatment. In tumor cells EGFR expression was found by IHC in 7 from 9 patients without gene amplification [36]. Therapy with both cetuximab and gemcitabine-oxaliplatin was suggested as palliative treatment in patients with advanced intrahepatic CC.

So far, there are no reports on the effects of cetuximab on growth inhibition in CC cell lines. The human CC cell lines used have been developed from different histological types and different stages of CC. EGI-1 was established from a bile duct carcinoma with advanced stage malignancy [37]. The initial tumor presented with seeded metastases and was histologically characterized as a large cell adenocarcinoma of low differentiation. HuH28 was established from liver bile duct carcinoma. OZ was established from ascitic effusion of a patient who suffered from obstructive jaundice due to the clogging of the common bile duct with mucinous substances produced by adenocarcinoma cells [38]. TFK-1 was grown from a surgically resected tumor (extrahepatic bile duct carcinoma) specimen, which had parts of a papillary adenocarcinoma and a differentiated tubular adenocarcinoma on histology [39]. EGFR expression was confirmed on all four CC cell lines by RT-PCR, Western blot and IHC. For EGFR this is consistent with data on other human CC cell lines: HuCCT1 express EGFR mRNA [40] and KMBC contain EGFR protein [41]. As inappropriate Ras activation is known to promote tumor cell proliferation we examined the four cell lines for *KRAS *gene mutations. In EGI-1 a heterozygous mutation was found. HuH28, OZ and TFK-1 were confirmed to display the wild-type allele. Constitutively activated Ras is associated with continuous growth stimulation. The cell lines EGI-1 and TFK-1 were chosen for growth inhibition experiments to further compare the two different *KRAS *genotypes. Cetuximab did not significantly inhibit cell growth in EGI-1 cells, but had a dose-dependent effect on growth of TFK-1 cells. This evidence suggests that the outcome of growth factor inhibitor treatment could be affected from the K-*ras *genotype.

Yoon et al found that EGFR activation was sustained following EGF stimulation in cholangiocarcinoma cells as compared to hepatoma cells [29]. They used KMBC and Witt cell lines. EGFR activation resulted in p42/44 MAPK activation. Cell growth was increased in cholangiocarcinoma following EGF stimulation and this was significantly attenuated by kinase inhibitors. To our knowledge, as yet, no study has compared the growth inhibitory effects of EGFR antibodies and kinase inhibitors. This group further found a defective receptor internalization in a CC cell line. However, they did not look into KRAS mutations. It would be of interest to study cells used by Yoon et al. with respect to their KRAS status.

As it was reported that cetuximab can induce apoptosis in tumor cells *in vitro *and *in vivo *[26,42,43], we tested the effect of cetuximab on cell survival. As we wanted to compare cell lines with activating KRAS mutation and without with respect to their response to cetuximab the cell lines EGI-1 (with activating KRAS mutations) and TFK-1 (without KRAS mutations) were chosen for growth inhibition experiments to further compare the two different KRAS genotypes. Cetuximab did not significantly inhibit cell growth in EGI-1 cells (containing the activating KRAS mutation), but had a dose-dependent effect on growth of TFK-1 cells. This suggests that the outcome of cetuximab treatment of CCC cell lines could be affected by the K-ras genotype. Therefore other modes of action might be involved in the effects observed on the growth of TFK-1 at least at lower cetuximab concentrations. The amount of EGFR present on the cell surface was not measured in our experiments. In colorectal cancer, there is no direct correlation between the amount of EGFR on the cell surface and the effects of EGFR blockade *in vivo*. And EGFR-negative colorectal cancer patients have been reported to respond to cetuximab treatment. From our data, it could be speculated that similar effects might be seen in CC. Further research is needed to demonstrate whether histology can predict a response to EGFR blockade in CC. Growth inhibition of cetuximab in TFK-1 cells was dose-dependent. This may imply that higher concentrations *in vivo *have more pronounced therapeutic effects. In contrast to cholangiocarcinoma in colorectal cancer, dose-escalating studies are on its way and their results are awaited.

More limitations apply to our study: we studied cell lines and it is inherent that the results may not be readily transferable to the *in vivo *situation. Moreover, although we used four different cell lines for the mRNA and protein expression experiments and two for the studies on the effect of cetuximab, these numbers are still low compared to the myriad of differences which should be found in different tumors in vivo. Nevertheless, the results of this study help to generate relevant questions for research in the treatment of cholangiocarcinoma. One of them is that KRAS mutations should be examined in the clinical trials on the effect of EGFR blockade in this tumor type.

In our experiments HGFR was detected in all four CC cell lines by RT-PCR, Western Blot and IHC. Others found HGFR mRNA in rat cell line CC-62, which was derived from a combined hepatocellular and cholangiocellular carcinoma [44]. HGFR expression is high in well-differentiated tumors and relatively low in poorly differentiated tumors [45]. Antagonizing the binding of HGF to HGFR also inhibited invasion in HuCC-T1, a human CC cell line, in vitro and in vivo [46] suggesting that blockade of HGFR might be a therapeutic strategy which should be the focus of further studies.

IGF1R expression was confirmed in the four CC cell lines tested by RT-PCR and IHC. Western blot showed protein expression in EGI-1 and TFK-1. Alvaro et. al. reported that IGF1R is expressed in the CC cell lines HuH-28, TFK-1 and Mz-ChA-1 [47] which supports our finding. IGF1R antagonists can inhibit proliferation of CC cell lines after serum deprivation and re-administration. In addition, IGF1R antisense oligonucleotides diminished cell growth in HuH-28 cells [47]. Increased expression of IGF1R promotes ligand-dependent malignant transformation in various cell systems [48]. To our knowledge, there have been no reports on the expression on IGF2R in CC cell lines or CC. In our experiments IGF2R mRNA and protein expression was found in EGI-1, HuH28, OZ and TFK-1 with all methods applied.

VEGFR1 expression was found by RT-PCR and IHC in the four CC cell lines tested but not with Western blot. To date, there is poor knowledge about the expression of VEGFR in CC. Benckert et. al. confirmed VEGFR1 in 15 of 19 tumor samples in human CC biopsies by IHC and in *in situ *hybridization [49]. It was suggested that a malignant phenotype is associated with increased VEGFR1 expression.

This study has several limitations. First, only CC cell lines were used. These cells lines may have gained additional mutations during the many passages under in vitro conditions. This may be a reason for the detection of VEGFR3 mRNA in the absence of detectable protein. Further, we only investigated the blockade of EGFR. Other growth factor receptors may be crucial for the growth of CC cells. In addition we regarded TFK-1 cells as an example of CCC cell lines with WT KRAS and did not study HuH28 and OZ cells. This all limits the impact of this study.

## Conclusion

The CC cell lines investigated in this study express EGFR, HGFR and IGF2R. Some also display IGF1R and VEGFR1-3. Cetuximab did not significantly inhibit cell growth in EGI-1 cells carrying a heterozygous *KRAS *mutation, but had a dose-dependent effect on growth of TFK-1 cells displaying the *KRAS *wild-type. Thus growth factor inhibitor treatment could be affected from the *KRAS *genotype in CC similarly to data in colorectal carcinoma. The expression of EGFR itself does not allow prognoses on growth inhibition by cetuximab.

## Abbreviations

BSC: best supportive care; CC: cholangiocarcinoma; DAB: diaminobenzidine chromogen; EGF: epidermal growth factor; EGFR: epithelial growth factor receptor; H&E: haematoxylin and eosin; HGFR: hepatocyte growth factor receptor; IGF1R: insulin-like growth factor 1 receptor; IGF2R: insulin-like growth factor 2 receptor; IHC: immunohistochemistry; *KRAS*: v-Ki-ras2 Kirsten rat sarcoma viral oncogene homolog; n.d.: not detected; RT-PCR: reverse transcription-polymerase chain reaction; 5-FU: 5-fluorouracil

## Competing interests

• LX The author declare that he has no competing interests

• MH The author declare that he has no competing interests

• WD The author declare that he has no competing interests

• SK The author declare that he has no competing interests

• TP The author declare that he has no competing interests

• MS-G The author declare that he has no competing interests

• EL The author declare that he has no competing interests

• FK The author has received salary by Merck KGaA for giving a scientific talk on the treatment of colorectal carcinoma.

• GR The author declare that he has no competing interests

## Authors' contributions

All authors read and approved the final manuscript.

LX, study concept and design, performance of experiments, acquisition of data, analysis and interpretation of data. MH, performance of experiments, acquisition of data, analysis and interpretation of data, revision of the manuscript. WD, performance of experiments, acquisition of data, interpretation of data, experimental support. SK, interpretation of data, experimental support. TP, performance of experiments, acquisition of data, interpretation of data, experimental support. MS-G, interpretation of data, experimental support. EL, critical revision of the manuscript, interpretation of data, important intellectual contribution. FK, study supervision, study design, critical revision of the manuscript, interpretation of data, important intellectual contribution. GR, study supervision, study design, critical revision of the manuscript, interpretation of data, important intellectual contribution.

## Pre-publication history

The pre-publication history for this paper can be accessed here:

http://www.biomedcentral.com/1471-2407/10/302/prepub
